# Remodeling of tumour microenvironment: strategies to overcome therapeutic resistance and innovate immunoengineering in triple-negative breast cancer

**DOI:** 10.3389/fimmu.2024.1455211

**Published:** 2024-12-10

**Authors:** Desh Deepak Singh, Shafiul Haque, Youngsun Kim, Ihn Han, Dharmendra Kumar Yadav

**Affiliations:** ^1^ Amity Institute of Biotechnology, Amity University Rajasthan, Jaipur, India; ^2^ Research and Scientific Studies Unit, College of Nursing and Allied Health Sciences, Jazan University, Jazan, Saudi Arabia; ^3^ Department of Obstetrics and Gynecology, Kyung Hee University Medical Center, Seoul, Republic of Korea; ^4^ Plasma Bioscience Research Center, Applied Plasma Medicine Center, Department of Electrical & Biological Physics, Kwangwoon University, Seoul, Republic of Korea; ^5^ Department of Biologics, College of Pharmacy, Hambakmoeiro 191, Yeonsu-gu, Incheon, Republic of Korea

**Keywords:** triple negative breast cancer, immunotherapy, tumor microenvironment, drug resistance, nanomedicine

## Abstract

Triple-negative breast cancer (TNBC) stands as the most complex and daunting subtype of breast cancer affecting women globally. Regrettably, treatment options for TNBC remain limited due to its clinical complexity. However, immunotherapy has emerged as a promising avenue, showing success in developing effective therapies for advanced cases and improving patient outcomes. Improving TNBC treatments involves reducing side effects, minimizing systemic toxicity, and enhancing efficacy. Unlike traditional cancer immunotherapy, engineered nonmaterial’s can precisely target TNBC, facilitating immune cell access, improving antigen presentation, and triggering lasting immune responses. Nanocarriers with enhanced sensitivity and specificity, specific cellular absorption, and low toxicity are gaining attention. Nanotechnology-driven immunoengineering strategies focus on targeted delivery systems using multifunctional molecules for precise tracking, diagnosis, and therapy in TNBC. This study delves into TNBC’s tumour microenvironment (TME) remodeling, therapeutic resistance, and immunoengineering strategies using nanotechnology.

## Introduction

**Figure 1:**
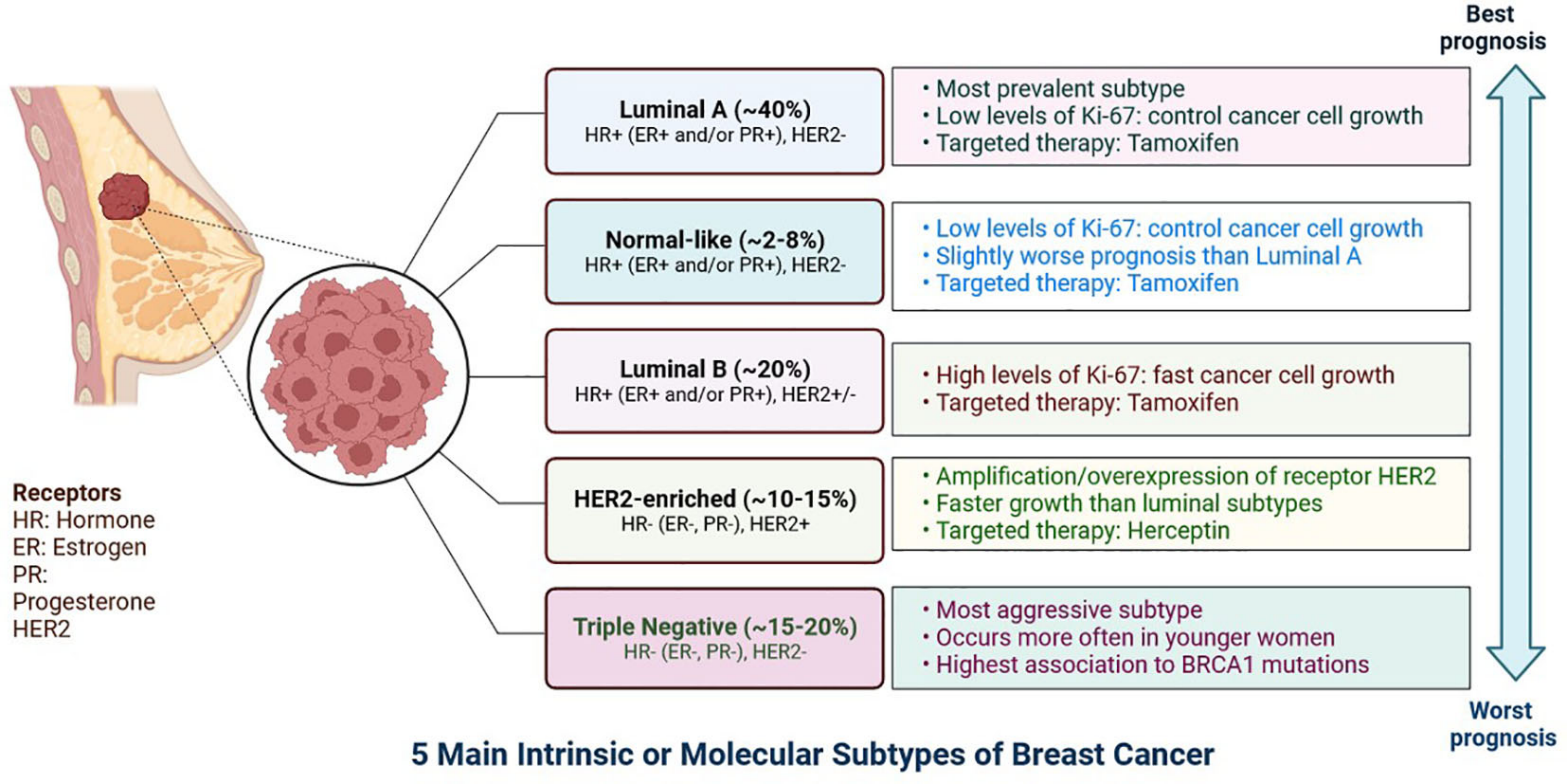
The expression of hormone receptors (progesterone receptor (PR) and oestrogen receptor (ER), the proliferation marker Ki-67, and the receptor tyrosine kinase HER2 can be used to classify breast cancer subtypes. Certain breast cancer subtypes can be treated with targeted medications like Tamoxifen, which targets the ER, and Herceptin, which targets the HER2 protein. The prognosis differs according on the kind of breast cancer.

Breast cancer is developing in the breast tissue. It is one of the most common forms of cancer in women but can also be found in men, albeit less frequently (1). Breast cancer can originate in various parts of the breast, including the milk ducts, lobules, or connective tissue (2). Triple-negative breast cancer (TNBC) is a highly aggressive subtype with significant intra-tumoral heterogeneity that often develops resistance to treatments (2). TNBC are classified into six subgroups: immunomodulatory, mesenchymal, luminal androgen receptor, basal-like 1, basal-like 2, and mesenchymal stem cells, based on the results of the transcriptome analysis (**Figure 1**). Tumour heterogeneity and a lack of biomarkers offer significant hurdles in overcoming treatment resistance and relapse (3).

The tumour immune microenvironment (TIME) is made up of a wide range of immune cells, as well as stromal cells, which contribute to its heterogeneity (3). The TIME composition changes throughout tumour growth and progression, as well as anti-tumor treatment (2, 3). In addition to the extracellular matrix (ECM), immune cells that are present in the tumour microenvironment (TME) and play a part in the development and spread of tumour necrosis include fibroblasts, antigen-presenting cells, and tumour-infiltrating lymphocytes (TILs) (4, 5).

Reprogramming the cellular physiology and avoiding immune damage are two recently identified signs of potential universality that have arisen in the last ten years (5). The body’s immune responses are enhanced and encouraged by tumour immunotherapy, which has the impact of eliminating tumours. It has emerged as an important anti-tumour therapy approach with significant clinical effectiveness and advantages over radiation, chemotherapy, and selective therapy. Current therapies used in clinical practice for the treatment of TNBC are shown in **Table 1** (6).

**Table 1:** Therapies used in clinical practice for the treatment of TNBC.

S. N.	Current treatment options	Molecular classification of TNBC	TNBC subtype
1	Anti-AR treatment (Bicalutamide, Enzalutamide)Anti-AR treatment (Bicalutamide, Enzalutamide)	High expression of the androgen receptor (AR) leads to increased hormone receptor signalling, including androgen/estrogen metabolism. High expression of the androgen receptor (AR) leads to increased hormone receptor signalling, including androgen/estrogen metabolism.	Luminal Androgen Receptor (LAR)
2	mTor inhibitors (rapamycin), growth factor inhibitors (lapatinib, gefitinib, and cetuximab),	Cell movement pathways are activated, extracellular matrix (ECM) interactions increase, and differentiation processes (Wnt and TGF-β) are dysregulated.	Mesenchymal (M)
3	Dasatinib is an inhibitor of Abl/Src, while Rapamycin inhibits mTOR.	Reduced cell proliferation and cell cycle gene expression, while increasing expression of stemness genes (HOX, NGF receptor, VCAM1).	Mesenchymal Stem Like (MSL)
4	Immune checkpoint drugs targeting PDL1 or PD1 include Atezolizumab and Pembrolizumab, as well as PARP inhibitors like Olaparib and Talazoparib. Platinum-based chemotherapy (cisplatin)	Genes related to immune cells (Th1/Th2 pathway, IL2 and IL7 pathways, NK cell pathway) are activated. Increased antigen presentation.	Immunomodulatory (IM)
5	Inhibitors of PARP olaparibm,Talazoparib, Platinum based chemotherapy cisplastin	T53 mutations and BRCA1 and BARCA2 mutation	Basal -Like -1 (BL1)
6	Inhibitors of PARP olaparibm,Talazoparib, Growth factor inhibitors (Lapatinib, Gefitinib and cetuximab)	Activation of EGFR,MET,IGF-1R,and Wnt/β- catenin signaling,	Basal -Like -2 (BL2)

Although a variety of tumour immunotherapeutic medicines have been developed, their broad use has been constrained by difficulties in their administration, including poor tumour permeability and low tumour cell absorption rates (7). Due to their targeting abilities, biocompatibility, and functions, nonmaterials have recently become a viable option for treating a variety of disorders (8). Additionally, nanoparticles have a number of qualities that make up for the shortcomings of conventional tumour immunotherapy, such as high drug loading capacity, effective tumour targeting, and ease of modification, which has led to the widespread use of nanomaterials in tumour immunotherapy (8). With the development of novel and potent therapies like checkpoint blockade therapy and CAR T-cell therapy that have significantly improved patient outcomes, immunotherapy has achieved clinical success in the past ten years. However, these treatments can be made more effective overall, reduce systemic toxicities, and reduce off-target effects (9). Different types of immune cells, such as dendritic cells for immunisation or T cells for enhancing adaptive immunity, can be targeted and modified by adjusting the nanomaterial’s features, such as size, shape, charge, and surface chemistry (10).

Currently, immunotherapy is a novel option for many solid tumours that have failed conventional therapies. There are various immunotherapeutic options available, including immunocheckpoint inhibitors (ICIs), inhibitors of cytotoxic T-lymphocyte-associated antigen 4 (CTLA-4) and programmed death receptor-1 (PD-1), as well as inhibitors of programmed death receptor-ligand 1 (PD-L1) (11). Due to its low tumour mutation burden and restricted T-cell infiltration, breast cancer has traditionally been regarded as a “cold” tumour. However, TNBC shows a larger amount of infiltrating lymphocytes, creating an advantageous immunological milieu for the possible application of ICIs (12).

## Remodeling of tumour microenvironment in TNBC

**Figure 2:**
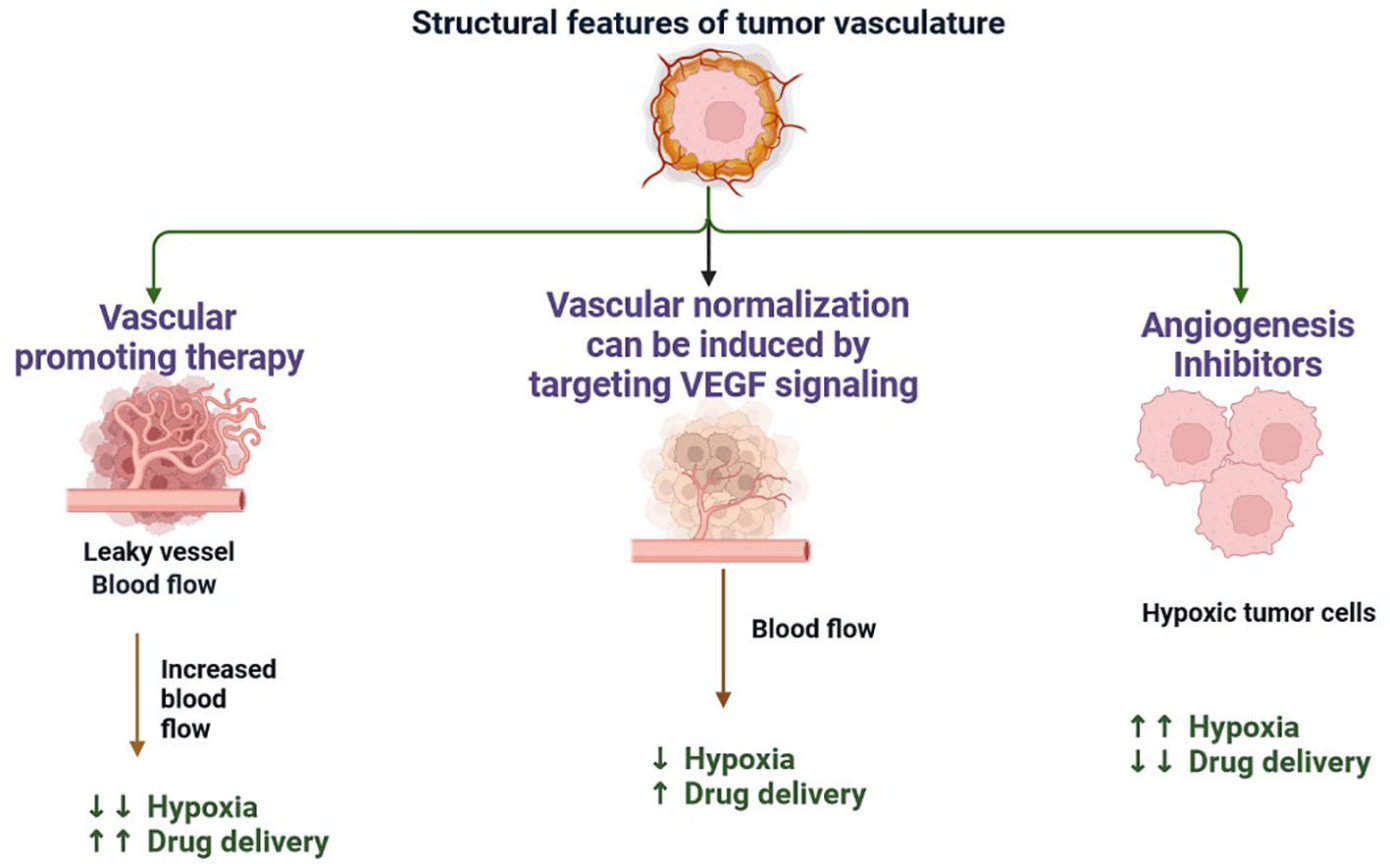
The formation of new blood vessels from the pre-existing vasculature is the main method of neovascularisation in tumours with their hypoxic and necrotic regions acting as inducers of angiogenesis. When a capillary receives an angiogenic stimulus, endothelial cell basal membrane and extracellular matrix are degraded, releasing endothelial cells from their basement membrane anchors (including integrins). This process is mediated by metalloproteases, leading to disruption of tight junctions, vasodilation, and pericyte detachment. Existing soluble growth factors coupled with the synthesis of a new matrix by stromal cells enable the migration and proliferation of endothelial cells.

**Figure 3:**
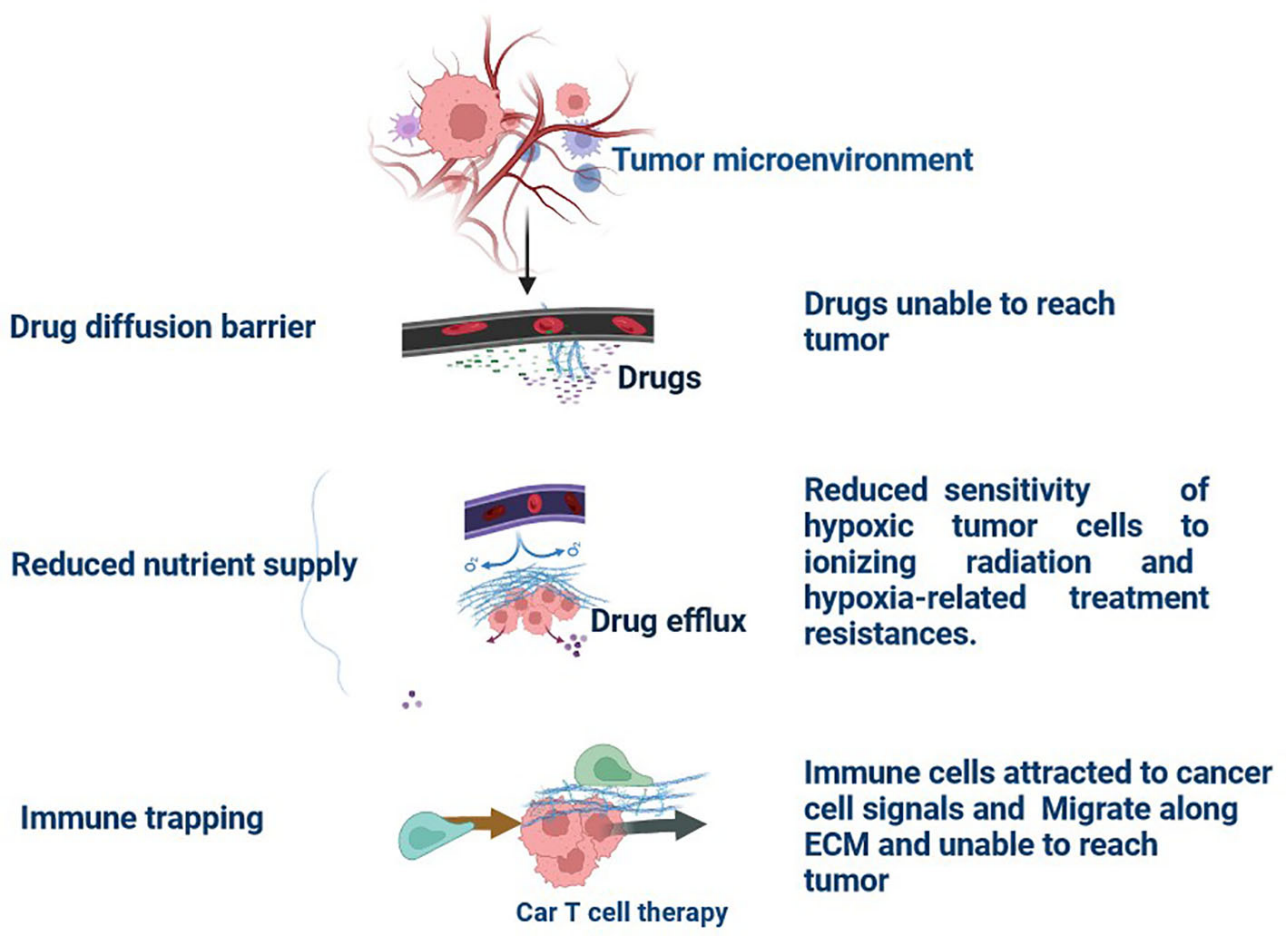
Tumour Extracellular Matrix Reduces Therapeutic Efficiency in TNBC: The tumour microenvironment (TME) comprises all components of a umor. Of these components, the extracellular matrix (ECM) is the least well studied. Solid tumours induce high expression of ECM molecules (collagens, proteoglycans, hyaluronic acid, and laminins), which become complex and disordered, resulting in altered haracteristics. Here, the ECM acts as a physical barrier, reducing the delivery of therapeutics, nutrients, and immune cells to solid tumours and leading to a poorer prognosis.

**Figure 4:**
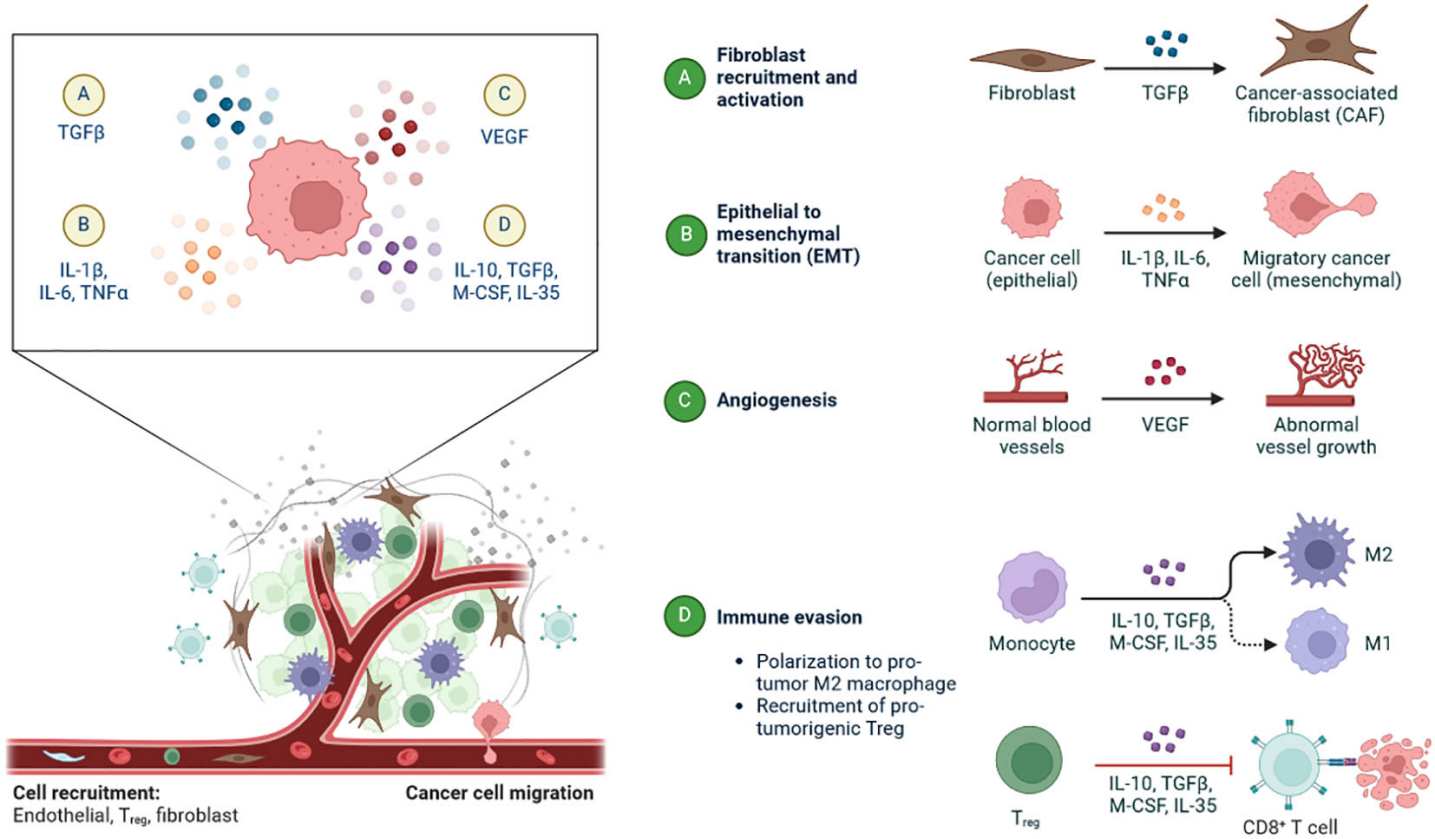
The tumour microenvironment plays a pivotal role in TNBC-associated changes: **(A)** Recruitment of fibroblast and activation by and regulates the TGFβ-TNBC-associated fibroblast (TAF). **(B)** Epithelial-to-mesenchymal transition (EMT) occurs in TNBC (epithelial) by IL-1β, IL-6, and TNFα-to Migratory cancer cells (mesenchymal). **(C)** Conversion of normal blood vessels into abnormal vessel growth by VEGF in angiogenesis. **(D)** Activation of M1, M2, and CD8+ T cells in immune invasion mechanism by IL-10, TGFβ, M-CSF, IL-35-M1 and M2 macrophases.

Multiple immunological and non-immune cell types contribute to long-lasting inflammation and localised immune suppression in this immune-modified milieu, allowing malignant cells to divide and mutate without being recognised and stopped by the host’s defense system (13). The TME is essential for the growth, spread, and therapeutic response of tumours (14). Creating cancer medicines that work requires an understanding of the TME (15). There is substantial variability in TME among the various subtypes of TNBC (16). These immune cells have the ability to aid immune evasion and tumour progression (17). Through the release of angiogenic substances, including vascular endothelial growth factor (VEGF), the TME can encourage angiogenesis (**Figure 2**) (18). Increased angiogenesis can feed the tumor with nutrients and oxygen, promoting growth and metastasis. There are various mechanisms by which tumours avoid immune recognition (19). TME is made up of different cell types, including fibroblasts, blood vessels, extracellular matrix elements (ECM), cancer cells, immune cells, and signalling molecules. Cancer therapy can be enhanced by modulating tumour blood vessels (**Figures 2**, **3**) (20). The ECM, an intricate web of proteins and glycoproteins, gives tissues structural support (21). The ECM’s composition and stiffness may change in TNBC, which may encourage tumour invasion and metastasis (**Figure 3**) (21). Fibroblast recruitment and activation contribute to the formation of a supportive stroma (22). Epithelial-to-mesenchymal transition (EMT) enables cancer cell invasion and migration (23). Angiogenesis ensures vascular endothelial growth factor (VEGF), nutrients, and oxygen supply to the growing tumour (24). There are various mechanisms by which tumours avoid immune recognition. In the absence of co-stimulation, tumour antigens are taken up and presented by APCs to tolerize T cells (25). Antibody against tumour cell-surface antigens induces endocytosis, degradation of the antigen, and immune selection of antigen-loss variants (25). Factors (e.g., TGFβ, IL-10, and IDO) secreted by tumour cells directly inhibit T cells, induce T regulatory cells, and generate the physical barrier to the immune system (**Figure 4**) (26).

## TAFs in remodeling of tumour microenvironment

TAFs (Tumour-Associated Fibroblasts) can activate in TNBC and release cytokines and growth factors that encourage tumour cell growth, invasion, and resistance to treatment (27). There are frequently areas of low oxygen (hypoxia) within the TME due to the TNBC tumours’ fast development (28). Angiogenesis, tumour aggressiveness, and therapy resistance can all be increased by hypoxia (29). There are six key approaches outlined, all with the aim of boosting the effector function of NK cells and helping to target cancer cells (30). Some, such as cytokines and immune checkpoint inhibitors, are clinically approved, while others are still in pre-clinical stages (31). Further work is needed to boost the efficacy of these treatments in solid tumours (**Figure 4**). In addition to affecting TAMs (Tumour-Associated Macrophages) and MDSCs (Myeloid-Derived Suppressor Cells). activating Natural killer (NK) cells is another approach being investigated for decreasing the immunosuppressive microenvironment (**Figure 4**) (7, 32, 33). The immunological activity of NK cells, a particular type of specialised innate lymphoid cells, is independent of MHC-mediated antigen presentation, Acts naturally as cytotoxic agents that inhibit the growth, migration, and colonization of metastatic cells, fighting both primary cancer cells and metastasis (34). Tumor Necrosis Factor Alpha (TNF-α), interferon-γ (IFNγ), and different interleukins are only a few of the cytokines that NK cells can release to control the immune response. They can also take part in other downstream immunological pathways (35). TAMs and MDSCs both contribute to the creation of an immunosuppressive microenvironment. T cells and NK cells can no longer act as immune cells when these cells are present (36). The nanoregulator can activate MDSCs to hasten the polarisation of TAMs towards the M1 phenotype and reactivate cytotoxic T cells to stop tumour cell proliferation. This further delays recurrence and reduces the chance that CTCs will establish themselves in the liver and lung thanks to the increased production of memory T cells that suppress cancer (37).

## Role of tumour microenvironment in Immune checkpoint inhibitors

The use of immune checkpoint inhibitors as a therapeutic approach to combat this immunosuppressive feature of the TME has been studied. High amounts of TILs are present in some TNBCs, which may indicate a better prognosis (38). Immunotherapies that stimulate TILs have demonstrated promise for treating TNBC. Targeted treatments and immunotherapies have been developed as a result of our growing understanding of TME remodeling in TNBC (39, 40). Chemotherapy and immunotherapy are ineffective primarily because of abnormalities in the TME that act as barriers to drug transport. TNBC tissue has significant ECM deposition and severe fibrosis, which causes tumour vascular compression and lowers perfusion, which impairs drug delivery (41). Cancer-associated fibroblasts (CAFs) release cytokines that influence the ECM, epigenetic alterations, immunosuppression, and proliferation of tumour cells (41).

TME remodelling (attracting CD4+T cells, CD8+T cells, and NK cells) enhances the effectiveness of immunotherapy, an essential approach used to treat breast cancer, as TILs in the TME have a direct link to the prognosis of TNBC (42). The targets for redesigning the TME have significance for the course of therapy and prognosis of TNBC because they affect TAMs and dendritic cells (DCs), as well as tumour hypoxia, tumour blood vessel modulation, CAF and ECM regulation, and CAF and ECM regulation (Figure) (43, 44). Through the release of cytokines, chemokines, and ECM remodelling factors, CAFs play a crucial role in promoting cancer progression and treatment resistance (27, 44–48). In order to kill cancer cells and sabotage CD47-SIRPa (CD47-signal regulatory protein alpha) connections, the hybrid cell membrane nanovesicles, or hNVs, can interface with circulating tumour cells (CTCs) in vascular lumens and accumulate at the site of resection (48). It has been found that gene enrichment of IFN-γ receptor, JAK2, and interferon regulatory factor 1 occurs in individuals who do not react to anti-CTLA-4 therapy.

**Figure 5:**
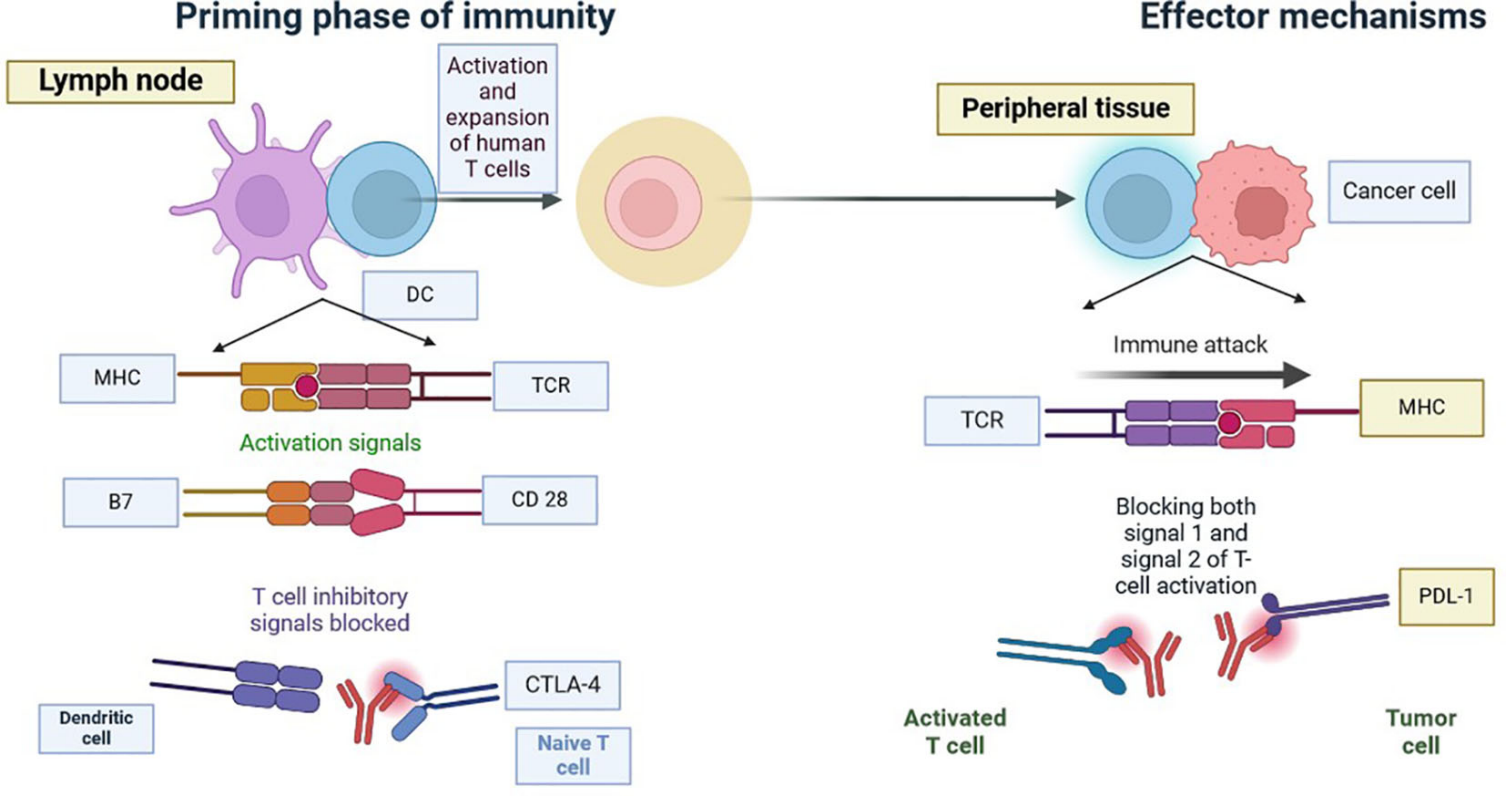
Blockade of CTLA-4 or PD-1 Signalling in Tumour Immunotherapy: Tumour immunotherapy has received a lot of attention since it has the potential to improve the immune system’s ability to recognise and fight cancer cells. Researchers hope to overcome tumours’ immune evasion strategies by suppressing CTLA-4 or PD-1 signalling pathways, thereby enhancing patient outcomes and extending therapy options. Tumour immunotherapy has received a lot of attention since it has the potential to improve the immune system’s ability to recognise and fight TNBC cells. To overcome tumours’ immune evasion strategies by suppressing CTLA-4 or PD-1 signalling pathways, thereby enhancing patient outcomes and extending therapy options.

**Figure 6:**
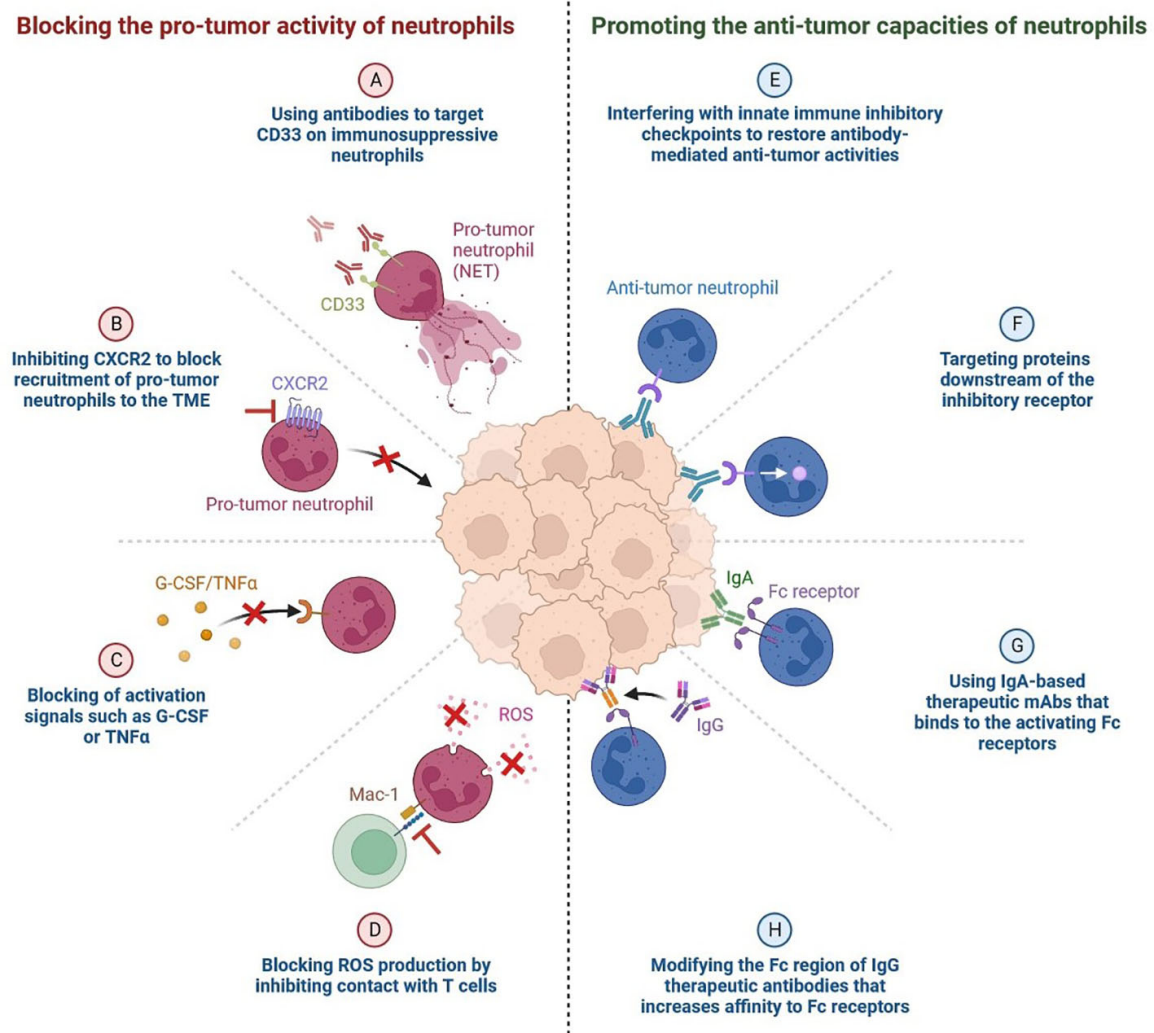
Targeting Neutrophils: The Pro- and Anti-tumor Activities of Neutrophils Blocking the pro-tumor activity of neutrophils. **(A)** Using antibodies to target CD33 on immunosuppressive neutrophils. **(B)** Inhibiting CXCR2 to block recruitment of pro-tumor neutrophils to the TME. **(C)** Blocking of activation signals such as G-CSF or TNFα. **(D)** Blocking ROS (Reactive oxygen species) production by inhibiting contact with T cells. **(E)** Promoting the anti-tumor capacities of neutrophils. **(F)** Interfering with innate immune inhibitory checkpoints to restore antibody-mediated anti-tumor activities.. **(G)** Targeting proteins downstream of the inhibitory receptor. **(H)** Using IgA-based therapeutic mAbs (Monoclonal antibodies) that binds to the activating Fc receptors.

The blockade of the PD-1 pathway brings quiescent antitumor T cells to active life (**Figure 5**). The pro-tumour activity of neutrophils is blocked by using antibodies to target CD33 in immunosuppressive neutrophils (49–51). CXCR2 (CXC chemokine receptor 2) is inhibited by blocking the recruitment of pro-tumour neutrophils to the TME, which blocks activation signals such as G-CSF or TNFα and ROS production by inhibiting contact with T cells (49, 52). We can enhance the anti-tumour capacities of neutrophils by interfering with innate immune inhibitory checkpoints to restore antibody-mediated anti-tumour activities, by targeting proteins downstream of the inhibitory receptor, by using IgA-based therapeutic mAbs that bind to the activating Fc receptors, and by modifying the Fc region of IgG therapeutic antibodies that increases affinity to Fc receptors, as shown in **Figure 6** (53).

## Adenosine and tumour microenvironment

**Figure 7:**
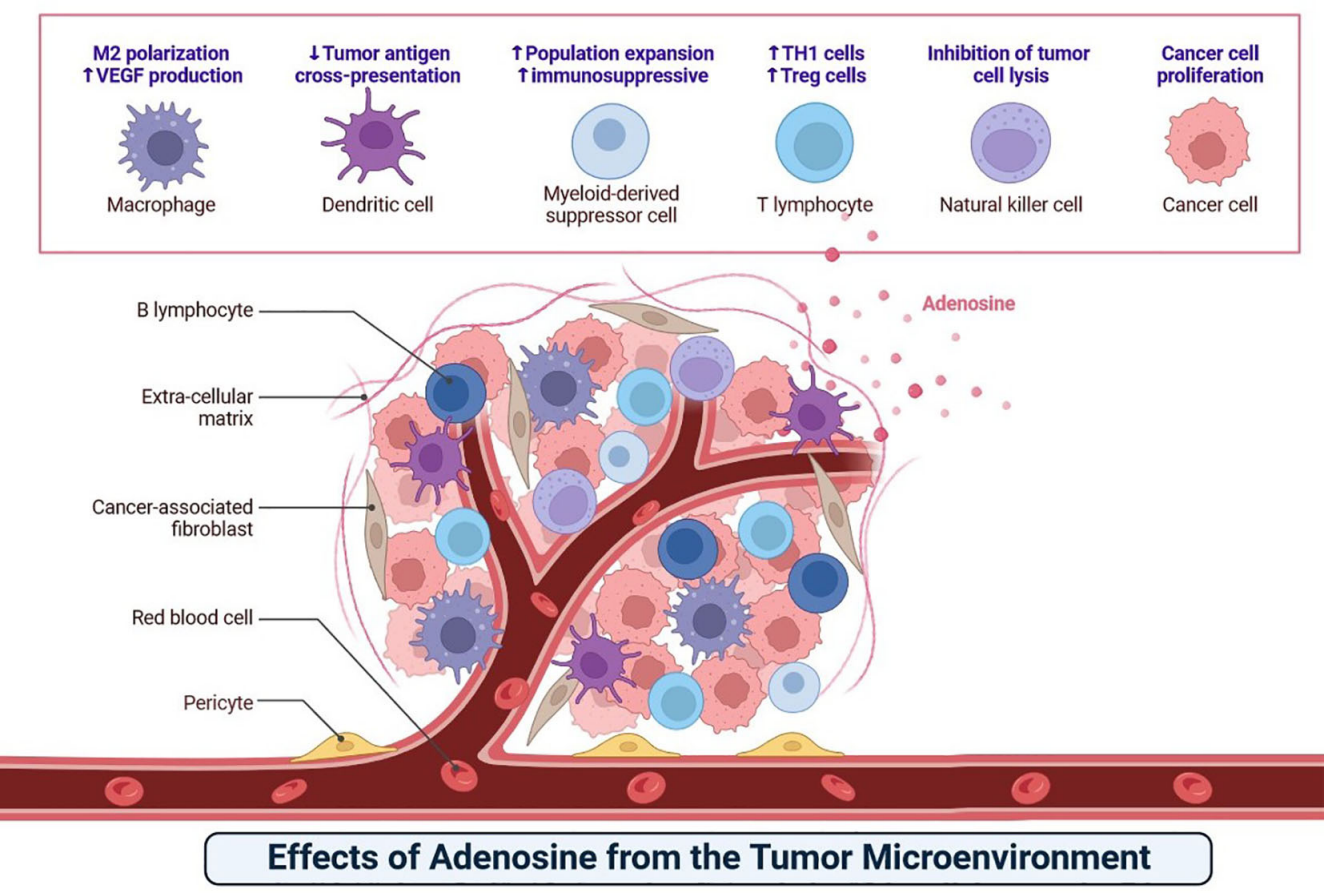
The adenosine causes immunosuppression that promotes cancer growth by interfering with B lymphocytes, T lymphocytes, natural killer cells, macrophages, dendritic cells, and myeloid-derived suppressor cells.

Adenosine, released by various cells within the tumour microenvironment, modulates local immune responses (**Figure 7**). This molecule impacts B lymphocytes, T lymphocytes, natural killer cells, macrophages, dendritic cells, and myeloid-derived suppressor cells, leading to immunosuppression that favours tumour growth (54). Understanding adenosine’s role in the tumour microenvironment provides a basis for identifying potential therapeutic targets for cancer immunotherapy (**Figure 7**) (53, 54).

## KRAS (Kirsten rat sarcoma virus) Mutations

**Figure 8:**
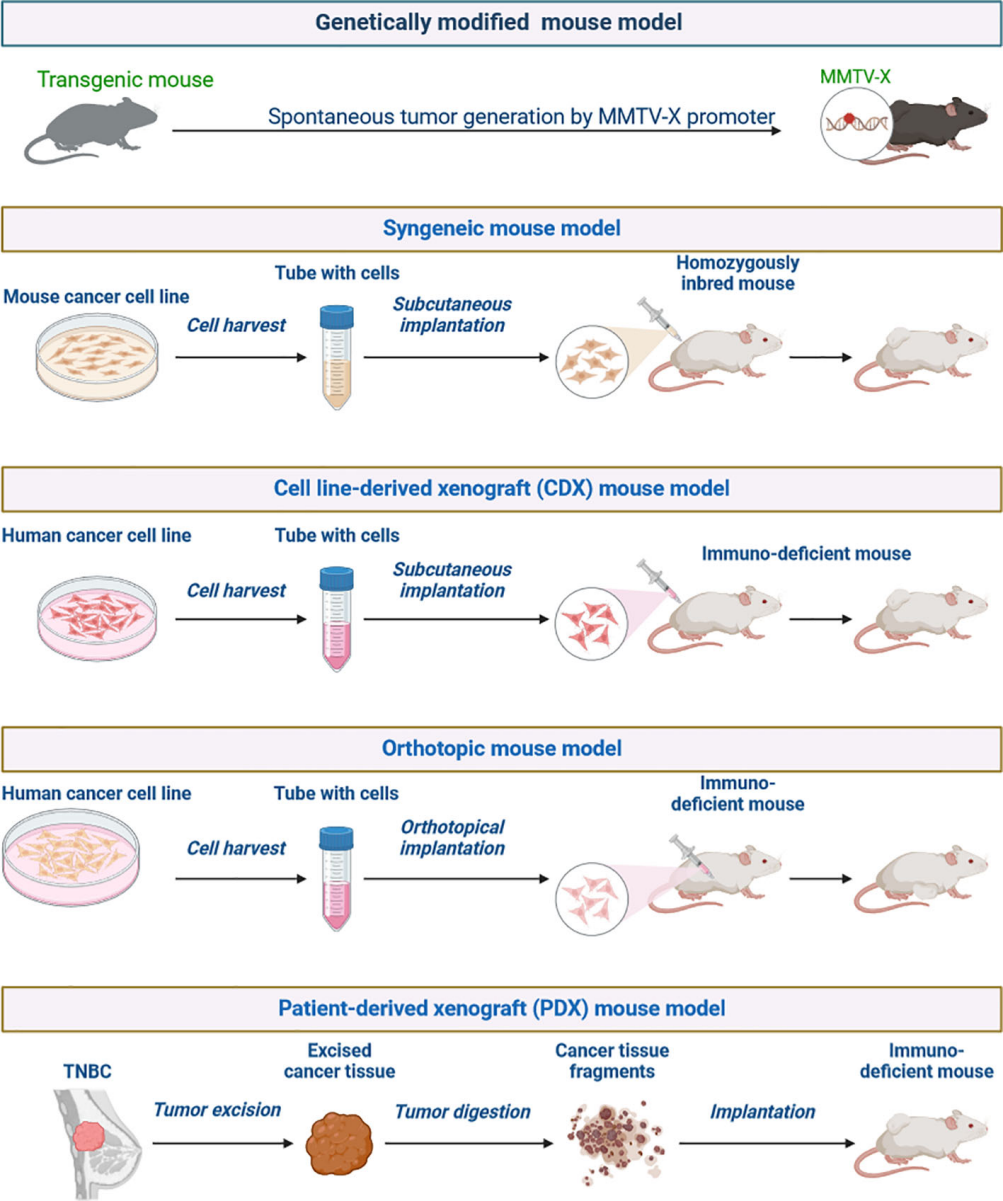
Generation of preclinical tumor mouse models for TNBC.

**Figure 9:**
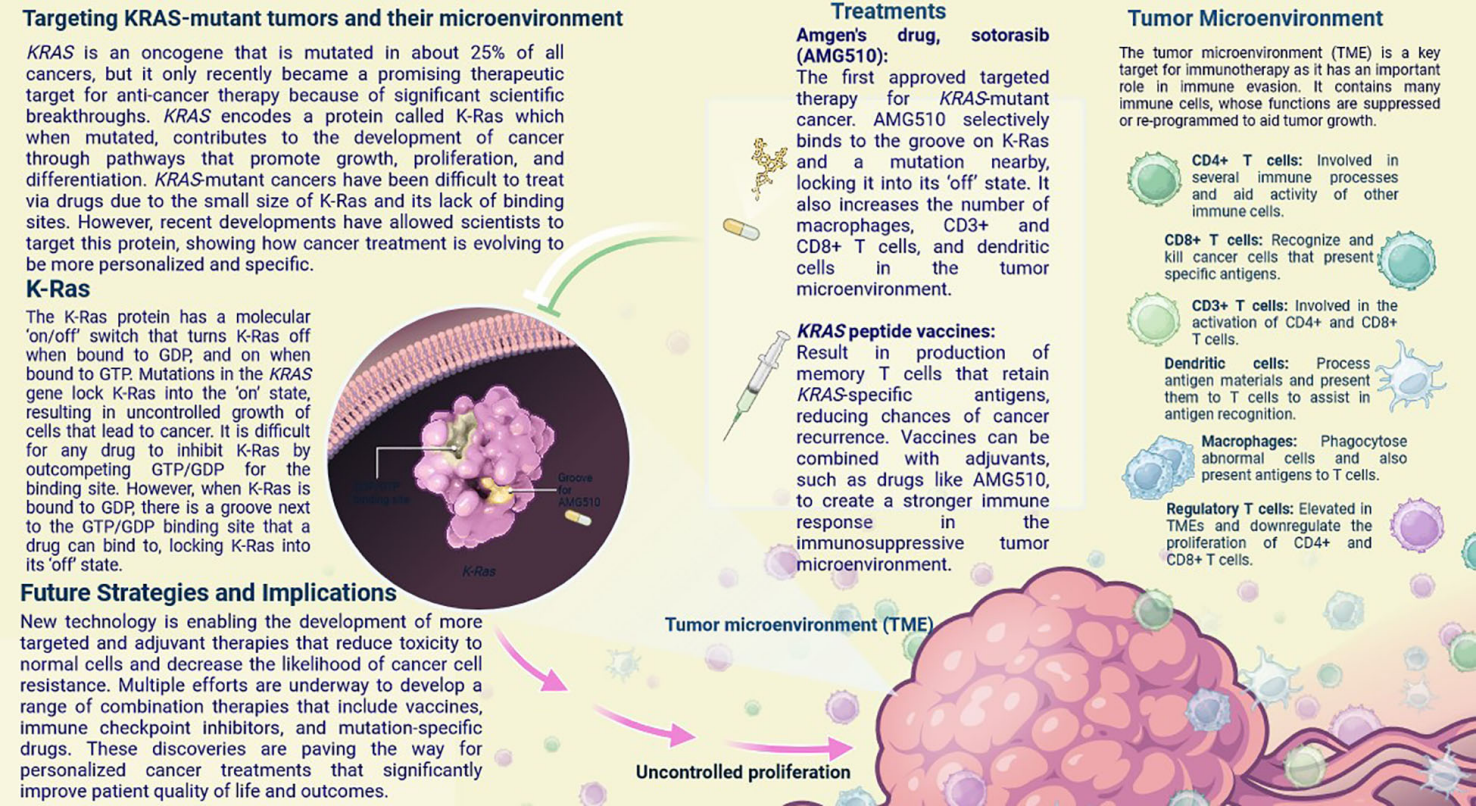
KRAS is an oncogene that is mutated in about 25% of all cancers, but it only recently became a promising therapeutic target for anti-cancer therapy. In this infographic, learn how recent breakthroughs have allowed scientists to develop treatments that specifically target mutant K-Ras, the protein product of KRAS, and alter the immunosuppressive tumor microenvironment to increase treatment efficacy.

KRAS (Kirsten rat sarcoma virus) is an oncogene that is mutated in about 25% of all cancers, but it only recently became a promising therapeutic target for anti-cancer therapy because of significant scientific breakthroughs (**Figure 8**) (1, 53, 55). KRAS encodes a protein called K-Ras, which, when mutated, contributes to the development of cancer through pathways that promote growth, proliferation, and differentiation. *KRAS*-mutant cancers have been difficult to treat via drugs due to the small size of K-Ras and its lack of binding sites (55). However, recent developments have allowed scientists to target this protein, showing how cancer treatment is evolving to be more personalised and specific. The K-Ras protein has a molecular ‘on/off’ switch that turns K-Ras off when bound to GDP and on when bound to GTP (55). Mutations in the *KRAS* gene lock K-Ras into the ‘on’ state, resulting in uncontrolled cell growth that leads to cancer. It is difficult for any drug to inhibit K-Ras by outcompeting GTP/GDP for the binding site (56). However, when K-Ras is bound to GDP, there is a groove next to the GTP/GDP binding site that a drug can bind to, locking K-Ras into its ‘off’ state (**Figure 9**) (57).

New technology is enabling the development of more targeted and adjuvant therapies that reduce toxicity to normal cells and decrease the likelihood of cancer cell resistance. Multiple efforts are underway to develop a range of combination therapies that include vaccines, immune checkpoint inhibitors, and mutation-specific drugs (57). These discoveries are paving the way for personalized cancer treatments that significantly improve patient quality of life and outcomes. The first approved targeted therapy for *KRAS*-mutant cancer (57). AMG510 selectively binds to the groove on K-Ras and a mutation nearby, locking it into its ‘off’ state. It also increases the number of macrophages, CD3+ and CD8+ T cells, and dendritic cells in the tumour microenvironment (58). Result in the production of memory T cells that retain *KRAS*-specific antigens, reducing the chances of cancer recurrence.

Vaccines can be combined with adjuvants, such as drugs like AMG510 (Sotorasib), to create a stronger immune response in the immunosuppressive tumour microenvironment (59). These exciting new developments show how cancer treatments are becoming more specific and personalised compared to classic cancer therapies like chemotherapy (57). The remodelling of TME can be achieved by either decreasing the matrix barrier or enhancing the immunosuppressive microenvironment, concentrating on the gut microbiota and metabolites, and modifying immune cells and cytokines (58, 59). These methods contribute to the improvement of TNBC anti-tumour therapy as an entire concept by using appropriate model (60).

## Preclinical models in mimic of TNBC

**Figure 10:**
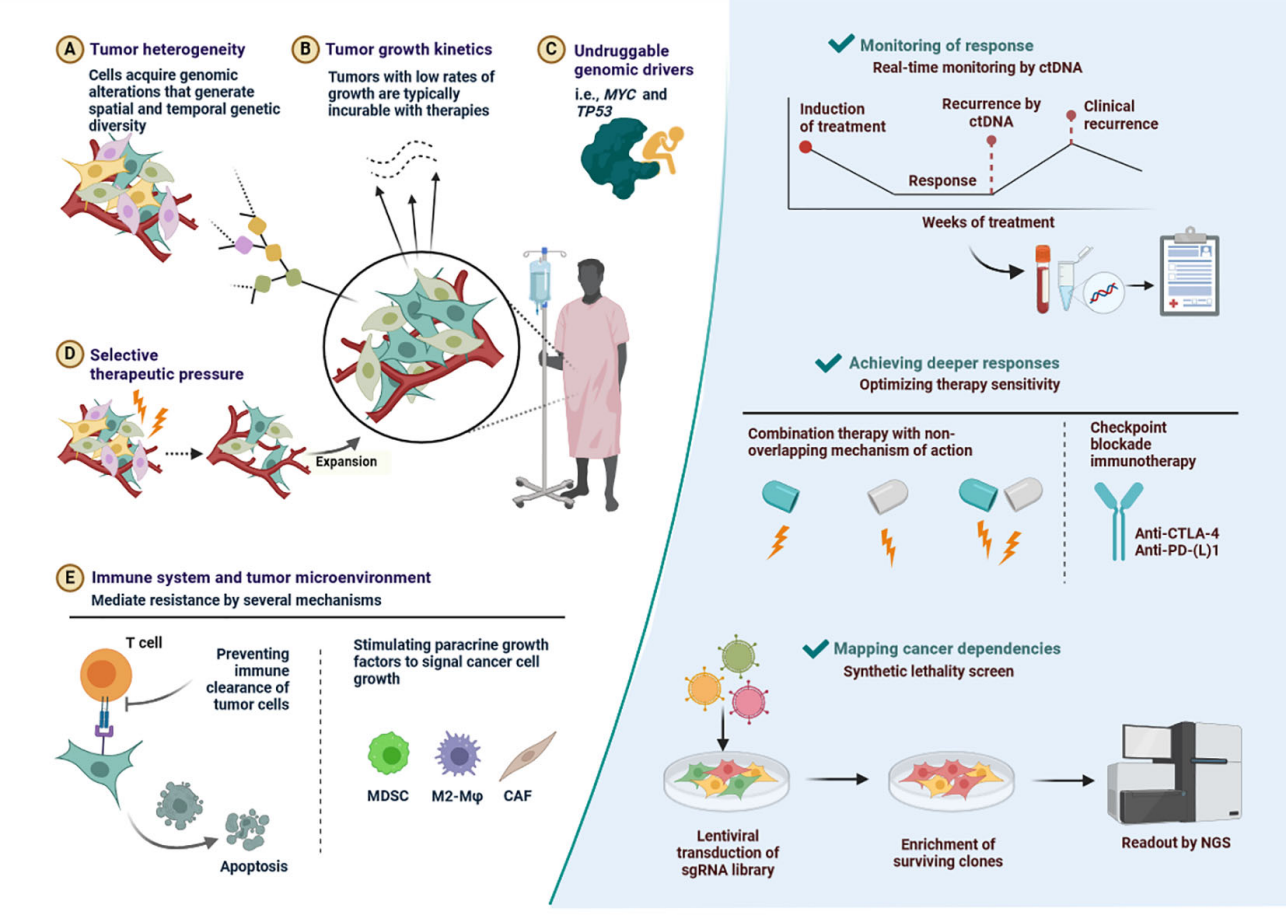
Key determinants of drug resistance as well as some potential, general solutions. **(A)** Tumor heterogeneity-Cells acquire genomic alterations that generate spatial and temporal genetic diversity. **(B)** Tumor growth kinetics-Tumors with low rates of growth are typically incurable with therapies. **(C)** Undruggable genomic drivers-i.e., MYC and TP53. **(D)** Selective therapeutic pressure-Expansion. **(E)** Immune system and tumor microenvironment-Stimulating paracrine growth factors to signal cancer cell growth.

Preclinical models that accurately mimic TNBC aetiology are vital for assessing new therapy options and identifying long-term benefits for patients (60). To maximise model predictive accuracy while conserving time and resources, novel therapeutic strategies are planned, taking into account preclinical studies and clinical trial expenditures. Individualised therapies for TNBC patients are in high demand to improve their future healthcare (60, 61). There is a unique opportunity to use preliminary models and techniques in laboratory studies. To study TNBC disease at lab work to identify key genetic, transcriptomic, and proteomic players in tumour initiation and progression and identify potential anticancer agents for precision medicine. An ideal preclinical TNBC model should have close histological similarities to the tumour, maintain druggable genetic changes for targeted treatments, and be easy to handle and grow well *in vitro* and *in vivo* (**Figure 10**) (60, 61).

## Novel breast cancer drugs in use or in clinical trials for the treatment of TNBC

Numerous possible agents are undergoing various phases of investigation and advancement. These possible agents/inhibitors carry out their anti-tumor activity (62). An overview of the various inhibitor classes, including poly (ADP-ribose) polymerase (PARP), tyrosine kinase (TK), EGFR, PI3K, heat shock protein (Hsp90), histone deacetylase (HDAC), angiogenesis, insulin-like growth factor (IGF), and mammalian target of rapamycin (mTOR), as well as the mechanism of action depicted. In summary, ssDNA break repair is the aim of poly (ADP-ribose) polymerase inhibitors (PARPI). An 88% overall response rate was observed with the combination of paclitaxel and olaparib (40, 63, 64).

Receptor tyrosine kinase (RTK) inhibitors have been investigated for treatment options in TNBC. Unexpectedly, the bulk of EGFR-TKI studies against TNBC are not encouraging, despite the fact that EGFR is expressed in 89% of TNBC and looks to be a viable therapeutic target (64). Since only around 10% of TNBC has FGFR (Fibroblast Growth Factor Receptors) identified as a therapeutic target, pan-FGFR inhibitors such alofanib and PD173074 prevent SUM52PE from proliferating and cause apoptosis by blocking the MAPK and PI3K signalling pathways (65). Although bevacizumab and apatinib, which target VEGF2 (Vascular endothelial growth factor), have not shown encouraging effects in clinical studies, VEGF expression is linked to a poor prognosis in TNBC. On the other hand, sunitinib, an anti-VEGFR (Vascular endothelial growth factor) tyrosine kinase inhibitor, is starting to show promise as a treatment option in breast cancer studies (66).

The TNBC cell surface RTK (Receptor tyrosine kinases) MET (Mesenchymal–epithelial transition) triggers the activation of many downstream effectors, including as Src, AKT, ERK, and RAS. The tivntinib (MET inhibitor) phase II study is disappointing; however MET+EGFR inhibition synergistically decreased cell viability, demonstrating the combination’s higher effectiveness (67). Patients with TNBC have participated in clinical studies to assess a variety of PARPIs, including olaparib, veliparib, and talazoparib. The phase III trial of olaparib for BRCA-mTNBC (OLYMPIAD; NCT02032823) will The cytoplasmic mic kinases known as non-receptor tyrosine kinases (NRTKs) include MEK (Mitogen-activated extracellular signal-regulated kinase), Src, and the PI3-AKT-mTOR signalling cascade (68, 69). When combined with everolimus, dual mTORC1/2 inhibitors effectively limit the growth of many TNBC cell lines. Ongoing clinical studies are being conducted on TNBC using dual mTOR/P13K inhibitors and mTOR+PARP inhibitors (69). Early in the clinical development process, the PI3-AKT-mTOR pathway is one of the growing multi-targets of pharmaceuticals. MEK is a part of the MAPK signalling cascade, and MEK inhibitors (U0126) have been shown to dramatically diminish the invasiveness of MDA-MB-2311 *in vitro*, while selumetinib has been shown to suppress lung metastasis in xenograft models (70, 71). The addition of Src inhibitors, such as dasatinib to cetuximab + cis platin, improved the prevention of cell growth and invasion in TNBC (71). Src is a cytoplasmin oncoprotein. HDACs (Histone deacetylases) and Hsp 90 are two examples of epigenetic targets that are being researched for the therapy of TNBC (72). Tumour suppressor and DNA repair gene expression are known to be inhibited by HDACs. HDACi’s (Histone deacetylase inhibitors) in combination with cisplatin and DNA methyltransferase inhibitors are being studied in two clinical studies (72).

It is possible for Hsp90 to block many growths, signalling pathways, and survival cascades. Although olaparib and paclitaxel are still being tested in phase 1 clinical trials, a Hsp90 inhibitor called genetecibo (Ganetespib) has been shown to decrease tumour volume in xenografts produced from MDA-MB 231 (73). The anti-androgens bicalutamide and enzalutamide reduce proliferation, invasion, and migration of cancer cells by targeting the androgen receptor (AR) in different TNBC cell lines (73). This suggests that they may serve as a surrogate biomarker for response to other treatments. The VGS subtype Nav1. 5 neonatal splice variant is known as voltage-gated sodium channel (VGSC) (74). The foundation of clinical care of TNBC is VGSC-inhibiting medications, which include phenytoin, ranolazine, and riluzole. These medications all reduce metastatic cell behaviours *in vitro* and/or *in vivo* (75).

NP-based formulations called Liposomes-Doxorubicin nanodrug and Myocet, which were licenced for the treatment of breast cancer in 1998 (Taiwan) and 2000 (EMA), respectively, are now being used in clinical settings to treat metastatic breast cancer (76). A multitude of nanoparticles, including liposomal and polymeric nanoparticle platforms, are being developed for the treatment of cancer. The mitoxantrone-containing liposomal nanoparticles known as plm60-s (Mitoxantrone HCl liposome injection) are undergoing a phase II clinical study for the treatment of breast cancer. In phase II of a clinical study, the LiPlaCisa liposome nanoparticle containing cisplatin is showing promising outcomes for metastatic breast cancer (76).

Numerous immunotherapies, including as immune checkpoint inhibitors, activation of cytotoxic T lymphocytes (CTLs), adaptive cell transfer-based treatment (ACT), and modification of the tumour microenvironment (TME), have been tried. These cutting-edge immune-modulatory techniques can treat TNBC and have become individualized immunotherapy as shown in **Table 2** (77).

**Table 2:** TNBC tumor microenvironment-based nanotherapy.

S.N.	Therapeutic agent(s)	Nanocarrier	Key outcomes	Reference
1	Doxorubicin	Amphiphilic copolymer micelles linked via β-thiopropionate bonding	Linkage is hydrolyzed by acids. After 100 hours, drug release was 80% at pH 5.2% and 35% at pH 7.4.	(99)
2	Doxorubicin	pH-sensitive tri block copolymeric micelles including peptides that penetrate cells	The release of doxorubicin was around 65% at pH 5.0 and 32% at pH 7.4.	(99)
3	Paclitaxel	pH-responsive liposomes	Paclitaxel releases faster at acidic pH and is more helpful to breast cancer models in both vitro and *in vivo* settings.	(101)
4	Doxorubicin	Glycolipid-like nanocarrier based on chitosan	Cytotoxic to MCF-7 breast cancer cells due to the 5extracellular environment	(102)
5	NS629	Stromal cells -lipidated cathepsin B inhibitor-coated liposomes	T6argeting cathepsin B which is all7owed for the selective targeting and internalization of liposomes, which improved both ex vivo and *in vivo* administration in TNBC model.	(103)
6	siRNA	Amphiphilic copolymer micelles linked via β-thiopropionate bonding	Tumour acidity caused the PeG surface layer to separate, allowing for easier cellular absorption. The hydrophobic PLGA layer caused siRNA to be released into tumour cells very quickly.	(104)
7	Docetaxel	Acetylated carboxymethylcellulose nanoparticles connected with PEG	Revealed reduced tumour growth and metastasis and increased MTD in many xenograft models in comparison to Abraxane; moreover, the 4T1 and MDA-MB-231 models displayed α-smooth muscle actin levels that were 82% and 70% lower, respectively.	(105)
8	Doxorubicin	FSH receptor on tumor vasculature with Graphene oxide nanoparticles containing FSH antibody	GO–FSHR–m vasculature accumulation Early-stage Ab conjugates in tumours; improved drug delivery efficiency in MDA-MB 231 metastatic locations	(106)
9	Carboxyfluorescein as fluorescent dye	MMP-9-Liposome with degradable lipopeptides	MMP-9-degraded lipopeptide, markedly increased release rate when MMP-9 is present	(106)

## TME and therapeutic resistance in TNBC

Various immune-promoting and immune-resistant components interact to form a complex network which regulates the TME (78). CTL interacts with APC and NK via cytokines and is essential to immunotherapy (51). Tumor-associated macrophages (TAM), myeloid-derived suppressor cells (MDSC), and regulatory T cells (Treg) are examples of immune-regulating cells (79). These cells impede the growth of T cells by directly expressing immunosuppressive cytokines and immunological checkpoint molecules. An immunosuppressive TME can also be produced by these cells’ malfunctions through indirect pathways including PD-1 pathway hijacking and interactions with Treg (80).

T cell infiltration is impeded by aberrant tumour neovascularization and cancer-associated fibroblasts (CAF). The TME becomes more acidic because of hypoxia-induced metabolic abnormalities, which creates a barrier to antitumor immune activity (81). Tumorigenesis generates aberrant antigens on the tumour cell surface that can activate APCs, mostly DCs. However, tumours frequently change the structure or downregulate MHC-I, which hinders antigen presentation and encourages immune escape (81).

TNBC patients that are resistant to ICB treatment frequently have downregulated levels of HLA-I, the human version of this molecule. This may be caused by mutations in the invariant β2-microglobulin (β2m) gene and other HLA-I encoding genes (51). The control of HLA-I expression is also dependent on transcription factors including NF-κB and NLRC5, as well as epigenetic processes, the disruption of which would have a major impact on antigen presentation (82). A prime example is interferon (IFN), which may upregulate HLA class I heavy chains (β2m, TAP1, TAP2, or Tapasin) and cause HLA-I to be downregulated when it is impaired (83). Furthermore, peptide antigen processing and presentation will be impacted by genetic flaws in any of the proteins that make up the MHC-I processing apparatus, such as downregulation of the TAP transporters. Important phases in the immune activation cascade involve APC activation and recruitment. Immune escape is supported by the reduction of chemokines which recruit APCs (84). In order to prevent APC phagocytosis, tumours can also suppress “eat me” signals like CRT and HMGB1, and upregulate “don’t eat me” signals like CD47, CD24, etc. When TNBC is present, the glycosylation of B7-H4 stabilises and stops this protein from degrading, which suppresses eIF2α phosphorylation (85). This results in a decrease in CRT surface expression and lets the tumour avoid immune destruction (85). It has been demonstrated that dendritic cells downregulate CD80 expression, preventing T cell activation via CD28, and exhibit high amounts of PD-L1. This is believed to be one of the mechanisms for the failure of ICB therapies.

In addition to gene mutations, epigenetic modifications such as DNA methylation, RNA interference, histone modification, etc., can serve as immune escape mechanisms (86). By inhibiting the production of immune genes, epigenetic changes may have a negative impact on tumour immunity. For example, loss of methylation may be the mechanism via which highly altered malignancies avoid immune responses and account for the seemingly contradictory observation that tumours with large chromosomal copy number changes have low antitumor immune activity. DNA methylation can also mute genes related to immunity (87).

Abnormal MAPK pathway activation is inextricably connected to tumour formation and progression as well as treatment resistance mechanisms in different types of cancer. Low TIL in the basal-like breast cancer subtype is associated with activating alterations in the Ras/MAPK pathway, such as shortening of NF1, amplifications of KRAS, BRAF, and RAF1, according to data from the TCGA database (88). In addition, MEK expression has some predictive value for TNBC patients’ overall survival (OS) and recurrence-free survival (RFS) following neoadjuvant treatment (89). The antigen-presenting molecules MHC-I and -II expression seem to be inversely correlated with MEK activity. The prevailing consensus explains this by stating that the Ras/MAPK pathway might impede the IFNγ-mediated inflammatory response, which in turn impacts the IFNγ-mediated production of MHC-I, MHC-II, and PD-L1, suppressing antigen presentation (90). The WNT pathway controls DC-mediated innate immunity. ATF3, a transcriptional repressor that stops CCL4 from being transcribed, can be activated by β-catenin. Lack of CCL4 reduces the effectiveness of ICB treatments by impeding both the infiltration and activation of CD8+ T cells as well as the stimulation of CD103+ DCs (91). Thus, cancer cells have the ability to immunoedit by down regulating or changing key molecules in the IFN-γ signalling cascade, which ultimately leads to immune evasion. Cancer cells have the ability to up regulate PD-L1 transcription and expression as a negative feedback mechanism (92). There is some overlap between IFN-γ and type I IFN’s roles in antitumor immunity. More research is necessary since it is unclear how they work, what kind of reaction is elicited, and which cell population is reacting (93).

PTEN deficiency can impact T cell recruitment and function, which can lead to ICB therapy resistance. The loss of PTEN causes immunosuppressive factors like VEGF and CCL2 to express more. It has been demonstrated that anti-VEGF antibodies increase T cell infiltration and activation within the tumour (94). Therefore, PTEN deletion probably reduces T cell invasion through unregulated VEGF expression. Drug resistance in cancer therapy is major cause of mortality. **Figure 10** has described outlined key determinants of drug resistance as well as some potential, general solutions.

Exogenous nanomaterials can boost host immune escape and enhance the efficacy of immunotherapy. Nano-immunomodulators have been developed to specifically target the immunosuppressive microenvironment in order to loosen this rigid state (95). These agents can re-open the immune system *in situ* and slow the growth of tumours. The major nano-immunomodulators are predicted to target the microenvironment and inhibit metastasis based on the ability of reactivated immune cells to migrate (95). The biomimetic technique greatly reduces the toxicity of nano-assisted immunotherapy when compared to conventional nonmaterials, increasing its safety. Overall, by reducing the immunosuppressive effects on tumor-associated immune cells, remodelling the immunological state of the tumour microenvironment offers a workable strategy for immunotherapy (96).

## Nanotechnology based Immunoengineering approaches

**Figure 11:**
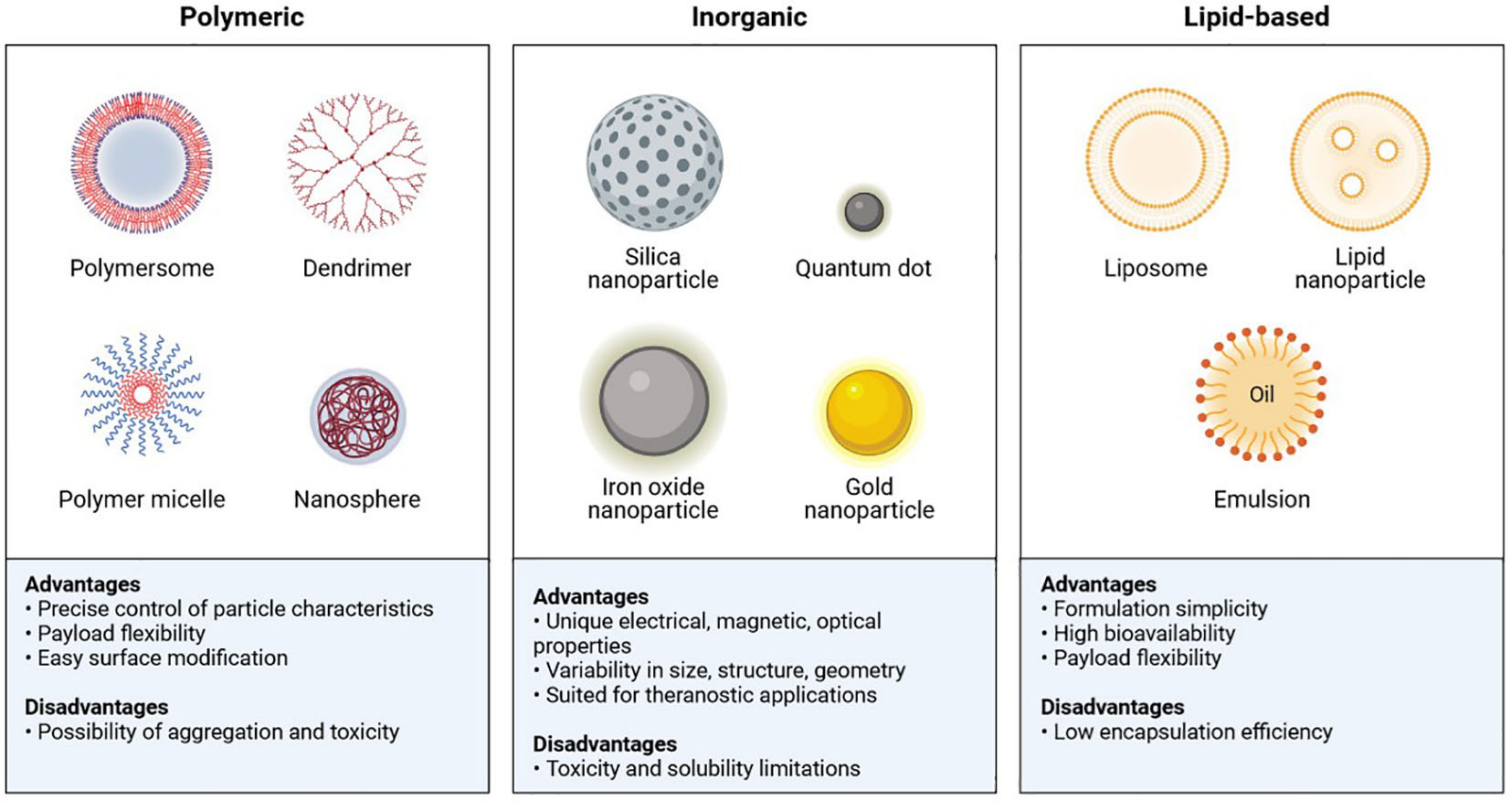
Main classes and features of Nanoparticles.

In the realm of nanoscience, a variety of physiochemical, biological, and functional characteristics are required to generate promising nanoparticles for use in biomedicine. The most crucial factor is size: conformational structure, targeted size (1 and 200 nm), high drug loading efficiency, a long half-life in circulation with minimal systemic toxicity, selective localization, and high adhesion in the tumour environment (97). There are various types of polymeric, inorganic, and lipid-based nanoparticles that, to their advantage, have been used against cancer cells (**Figure 11**) (98).

Gold nanoparticles (GNPs) have been shown to enhance radiation cytotoxicity in human cancer cells. Increased absorption of GNPs by cells may lead to greater radiation effects. We studied the radiosensitization effect of glucose-capped GNPs (16 nm and 49 nm) on MDA-MB-231 cells under megavoltage X-rays. Electron microscopy revealed that Glu-GNPs are primarily found in cells’ cytoplasm, including endosomes and lysosomes. ICP-AES shows that MDA-MB-231 cells absorb more 49-nm Glu-GNPs than 16-nm Glu-GNPs (P<0.05) (98). A lipid-based nanoparticle was chemically coupled to an inhibitor of the ECM-related enzyme, lysyl oxidase 1 (LOX), which inhibits the crosslinking of elastin and collagen fibres, resulting in a novel nanotherapeutic targeting the ECM. Conjugated vesicles loaded with chemotherapy-therapeutic epirubicin showed improved inhibition of TNBC cell proliferation *in vitro* and *in vivo*. Compared to free epirubicin and epirubicin-loaded nanoparticles, the *in vivo* results showed increased survival, reduced cytotoxicity, and improved biocompatibility (99). Using layer-by-layer films to adapt a liposomal doxorubicin delivery design with a synergistic siRNA can significantly reduce tumours in TNBC (100). Nevertheless, the use of nanomedicine in TNBC is restricted by the lack of ligands and a known highly expressed tumour target. Tumour microenvironment-based nanotherapy is described in **Table 2**, and strategies for therapeutic drugs via nanocarriers are shown in **Table 3** (100).

**Table 3:** Co-delivery strategy via nanocarriers for improved anticancer effects.

SN.	Therapeutic agent(s)	Nanocarrier	Key outcomes	
1	Rapamycin as chemotherapy agent; piperine as chemosensitizer	PLGA nanoparticles	Piperine increased the absorption of P-glycoprotein substrate rapamycin into breast cancer cells, resulting in a 4.8-fold increase in bioavailability.	(107)
2	Paclitaxel for chemotherapy; curcumin	Lipid nanoparticles conjugated with folate Cationic peptide	Increased paclitaxel and curcumin absorption in MCF-7/ADR cells	(108)
3	pTRAIL as a chemosensitizer and doxorubicin as a chemotherapeutic drug	Cationic peptide	MCF-7/ADR cells showed an 83.7% increase in cellular apoptosis along with a 94.0% tumour inhibitory rate; a synergistic effect was also seen.	(109)
4	Doxorubicin for chemotherapy; resveratrol to help	poly (lactic-co-glycolic acid) nanoparticle	Substantial *in vivo* tumour growth suppression with low toxicity; downregulated nuclear factor-κB and BCL-2 expression; inhibited expression of P-glycoprotein, MrP-1, BCRP, and triggered apoptosis.	(110)
5	Chemotherapy with doxorubicin and autophagy inhibition with chloroquine	Liposomes	IC50 in MCF-7/ADR cells was 5.7 times lower than that of free doxorubicin; in the spheroid and transgenic zebrafish models, it demonstrated superior anticancer effects compared to liposomal doxorubicin or doxorubicin alone.	(111)

## Versatile liposomal nanocarrier

Liposomes are 400 nm spherical vesicles, lipid bilayer, versatile nanocarriers, offering the effective drug distribution since they may encapsulate medications in either a lipid membrane or an aqueous core (111). Three different processes are commonly used to manufacture liposomal nanoparticles: reverse phase evaporation, solvent injection (which precipitates lipid from a dissolved lipid in solution), and extrusion (which produces nanoparticles with a predetermined cross-sectional area) (112). The dual drug targeted method has shown better outcomes in TNBC xenograft mice, liposomes containing sorafenib and doxorubicin have been shown to have increased antitumor efficaciousness. Clinical trial has been completed on a novel micelle-encapsulated doxorubicin formulation (NK911) with high efficacy and minimum toxicity (112). Antagomir-10b, an anti-metastasis agent, and PTX (Paclitaxel), an anti-cancer agent, were co-delivered via a liposomal drug delivery method to reduce lung metastases of breast cancer and limit the growth of 4T1 tumours (113). The lipid-conjugated estrogenic (bioactive; 47.03%) NPs were seen to display a substantially greater, or 87%, reduction of breast cancer development in engrafted mice (MDA-MB-231 cells) (113). Thus far, EndoTAG-1 and MM-398, liposomes laden with paclitaxel and irinotecan, have progressed to clinical trials in patients with TNBC (112).

## Micelles

Micelles are hydrophobic particles with a hydrophobic core formed by Vander Waals bonding, and they range in size from 5 to 100 nm. They are stabilized by a hydrophilic shell (114). Micelles have the ability to transport hydrophobic and water soluble drugs for the treatment of cancer due to their amphiphilic nature. Using styrene-co-malefic acid (SMA) (115). Researcher has synthesized an amicellar system to deliver RL71, a hydrophobic derivative of curcumin, for TNBC treatment. Due to a prolonged release profile and higher cellular absorption mediated by endocytosis, the system exhibited greater toxicity to cancer cells (115). Another synthesized cetuximab-conjugated vitamin E D-alphatocopheryl polyethylene glycol succinate to deliver docetaxel medication selectively (115).

## Dendrimers

Dendrimer was tested as a targeted diagnostic module in a TNBC cancer mouse model.A novel dendrimer, G4PAMAM, was generated and subcutaneously injected into mice as a dual model for imaging and drug delivery (66). It was combined with DL680 (NIR dye) and GdDOTA (MRI contrast). A near-infrared (NIR) fluorescence imaging scan and an MRI scan demonstrated the targeted diagnostic usage of this small sized (GdDOTA) 42-G4PAMAM-DL680 (GdDOTA) may be employed for certain diagnostic applications (116).

## Polymeric nanoparticles

Misnomer nanoparticles are polymeric nanoparticles (50 nm and 10 μm in size), These nanoparticles have the added benefit of encapsulating medications and proteins without requiring any structural modification. They can be made from natural or artificial polymers (117).

The combination of succinobucol and P188 (poloxamer) is showing promise as the best oral therapy for breast cancer. Succinobucol NPs have a better bioavailability (13 fold), which amplifies their capacity to prevent tumour cell migration and vascular cell adhesion molecule-1 (VCAM-1) invasion (118). Moreover, miRNA and siRNA have been shown to be delivered by polymeric NPs in conjunction with therapeutic drugs to reduce the tumour (119). Antisense-miR-21 and antisense-miR-10b were co-delivered by PLGA-b PEG polymer NPs at a concentration of 0.15 mg/kg, whereas co-loaded DOX and siRNA (multidrug resistance protein) induced an 8-fold decrease in tumour volume and growth overall, respectively (119). In TNBC tumour models, a potential ligand called Arg-Gly-Asp (RGD) either helps with targeted drug delivery or suppresses malignant invasion in a different way. For example, alphavbeta 3 (αvβ-3) integrin receptor adhesions and invasion have been demonstrated to be inhibited by cyclic RGD-functionalized solid lipid NP (RGD-SLN), which is overexpressed in invasive TNBC tumours (120). RGD-DMPLN’s targeted treatment efficacy was evaluated in a metastatic TNBC mouse model that was optimized with the MDA-MB-231-lucD3H2LN cell line. The total effectiveness of treating cancer is increased by this kind of targeted distribution of synergistic drugs; further research is needed to enable broader applicability in the case of breast cancer (120).

## DNA nanostructures in TNBC therapy

DNA nanostructures are designed with required forms, sizes, and configurations, such as tetrahedral, bipyramids, cages, and cubes, by taking use of the most fundamental property of DNA, which is Watson-Crick complementary nucleic acid base pairing (121). These DNA nanostructures may include tiny functional molecules and/or ligands for bioimaging or site-specific attachment (121). It was tagged at the base with red-emissive glutathione protected gold nanoclusters (GSH-Au NCs) and included actinomycin (AMD) in the minor groove of the DNA (122). Cetuximab’s has found effective in EGFR over expressing cancer cells, which is conjugated TH (THC3) with intercalated doxorubicin (DOX) medication, or THDC3, demonstrated preferential death of MDA-MB-468 cancer cells (122). The low IC50 value of 0.91 μM for THDC3 relative to 3.06 μM for free DOX indicates the strong and specific killing effectiveness of THDC3. Due to improved absorption of Cy3 THC3 into MDA-MB-68 cells, a different modified formulation comprising one Cy3 probe and three cetuximab, or Cy3-THC3, exhibits high signalling intensity (122). These two (THDC3 and Cy3-THC3) modest TH alterations demonstrate improved cancer cell targeting and killing, making them a great option for cancer nanomedicine, particularly for TNBC (122).

## Multifunctional metal nanoparticles

Metallic nanoparticles (NPs) like titanium dioxide (TiO2), gold (Au), silver (Ag), platinum (Pt), zinc (ZnO) are employed in cancer treatment. These nanoparticles’ magnetic, optical, thermal, and electrical characteristics may present several opportunities in therapeutic and diagnostic assays (123). The usefulness for intended therapeutic results is increased when metal nanoparticles’ surfaces are modified by conjugating distinct groups. NPs from the transition class of metals heat up the cells by converting electromagnetic energy to heat, causing hyperthermia (a non-invasive technique) that kills the tumour cells. Some metal nanoparticles possess distinct physiochemical characteristics that confer intrinsic strong anti-cancer action (124).

The most promising metal nanoparticle (NP) now available for delivering the well-known anti-cancer medication paclitaxel is gold nanoparticles (AuNPs) (125). Serum-coated AuNR has the innate capacity to suppress the expression of genes linked to energy production. Both *in vitro* and *in vivo* cancer cell migration and invasion are hindered as a result of decreased energy (125).

The cisplatin-coated AuNR in combination with an NIR laser has shown to inhibit/suppress the TNBC tumour and its metastasis. The antiproliferative, proapoptotic, and anti-angiogenic properties of silver nanoparticles (Ag NPs) on cancer cells are well-known (125). As a radiosensitive drug, AuNPs interacts with the acidic environment of cancer cells to enhance oxidative stress through the generation of reactive oxygen species (ROS), which ultimately cause damage and death (124, 125). The researchers have revealed encouraging outcomes from treating gliomas with AgNPs and then radiation. Additionally, it was shown that these NPs prevented cancer cells from expressing endothelial growth factor (VEGF), which reduced metastasis (126).

Zinc oxide nanoparticles (ZnO NPs) are used in cancer therapy and have even been shown to decrease toxicity and boost effectiveness in breast cancer cells when used in conjunction with the medications paclitaxel and cisplatin (127). In addition to copper (CuO NP), iron oxide (Fe2O3), silica, cerium oxide, and titanium oxide, other metal nanoparticles (NPs) are also being investigated and employed in the detection and treatment of breast cancer (127).

## Carbon nanotubesin TNBC therapy

Carbon nanotubes (CNTs) are folded into single-walled or multi-walled cylindrical structures. A slight modification in chemical change may perform a variety of tasks and has great potential for cancer treatment (128). Single-walled nanotubes (with a diameter of 1-2 nm) that may enter cells have both localised and sustained effects. By decreasing the number of macrophages and blood vessels within the tumour, oxidised multi-walled carbon nanotubes (o-MWNTs) offer a unique strategy for cancer treatment (129).

## Aptamers nucleicacid based ligands

Short oligonucleotide segments of single-stranded DNA or RNA are known as aptamers. Because of the aptamer’s distinct three-dimensional confirmation, it binds the target molecule with great affinity and strength (130). The degradation by nucleases is the sole restriction, yet its great stability attracted interest for the creation of molecular probes. The cell-SELEX approach has been used to precisely target a surface membrane protein on TNBC tumours, utilising the recently discovered LXL-1 aptamer (130). Using PDGF-aptamer coated with gold nanoparticles, the differential overexpression of the platelet-derived growth factor (PDGF) receptor in the TNBC cell line has been found. Mammaglobin A2 and B1 are overexpressed in breast cancer cells MCF7 and MDA-MB-415, according to observations (131). Terahertz (THz) chemical microscopy (TCM), which employs THz radiation, was used to find metastatic breast cancer using MAMA2 and MAMB1 aptamers. The nucleo-lin receptor in TNBC theranostic in certain breast cancer cells is exclusively bound by a unique 26-mer G-rich DNA aptamer; nevertheless, further research and combination with medication administration are needed for such an aptamer-based precision targeted diagnostic (132).

## Antibodies conjugated fluorescent nanoparticles

Antibodies are Y-shaped proteins with two epitopes that bind to their receptors with affinity and specificity (133). These are considered to be the best class of ligands for targeting.After conceptualising the differentially up-regulated expression of urokinase plasminogen activator receptor (uPAR) and tissue factor (TF) receptor in TNBC, researchers have proposed and confirmed the use of an anti-TF antibody labelled with copper-64 (anti-TF-antibody-64Cu) in an *in vitro* model of TNBC through PET imaging (134). Fluorescence microscopy and ultrasonography are also used to identify anti-EGFR and anti-VEGFR antibodies conjugated with fluorescent NP and ultrasonic contrast agents. Preclinical investigation on TNBC xenograft mice has demonstrated a satisfactory response (treatment) to Iodine-124 (124I)-tagged B-B4 antibody (targeting syndecan-1; CD138 antigen) and a good visualisation of the TNBC tumour (134–155).

## Peptides

Peptides are ligands with a low molecular weight that have a high degree of selectivity for intracellular activities. Chlorotoxin, RGD, P-selection, and tumour metastasis targeting (TMT) are a few peptides that are effective in targeting metastatic breast cancer (135, 156–173). By using NIR fluorescence imaging in breast cancer models of TNBC mice. CK3 peptide (Cys-Leu-Lys-Ala-asp-Lys-Ala Lys-Cys) binding to neuropilin 1 (NRP-1 transmembrane protein) (136, 174–201). Activated cell-penetrating peptides (ACPPs), which target the matrix metalloproteinase (MMP)-2 enzymes, increase tumour uptake and contrast imaging in *in vivo* tumour necrosis models when covalently attached to cyclic-RGD peptide (137). Better and more efficient targeting of αvβ3 integrin receptors was achieved by connecting modified Fe2O3 NPs to cyclic RGD peptides (138). PGD-peptide and P-selectin, when combined with liposomal NP, have the ability to bind to different tumour areas by overexpressing their respective receptors on breast cancer cells (139).

## Other small molecules

The possible targeted agent for cancer imaging has potential for direct imaging agents such as 18F-FDG, which is used as a glucose analogue. Studies have revealed that the folate molecule drives the super-paramagnetic iron oxide contrast agent (P1133) to folate receptors and internalises it in actively developing TNBC (140, 202–230). Even folic acid-conjugated AuNR demonstrated increased absorption in 4T1 metastatic breast cancer cells and targeted the folate receptor (141). Both quantum dots (QDs) and carbon dots (CDots) have shown potential in imaging and early detection of TNBC (141). TNBC development and metastasis are mediated by the cellular target chemokine receptor type 4 (CXCR4) (141). By increasing the cellular absorption into MDA-MB-231 cells, plerixafor or AMD3100 (CXCR4 ligand) conjugated poly(lactide-co-glycolide) NPs improved siRNA-mediated gene silencing. Similarly, in a lung metastatic model of breast cancer, AMD3100-loaded human serum albumin-encapsulated NPs target CXCR4 (141, 231–242).

Due to the strong affinity of hyaluronic acid (HA) for the CD44 receptor, MDA-MB-231Br (a kind of metastatic breast cancer cell) may take up an ultra-small (~5 kDa) HA-PTX nanoconjugate through CD44 receptor-mediated endocytosis (142). Tumour inhibition was much stronger when the urokinase plasminogen activator receptor (uPAR) targeting peptide was attached to poly (lactic-co-glycolic acid)-b-PEG polymers containing two antisense siRNAs. Novel contrast agents in MRI have been employed, using functionalized fullerenes (143, 243–250). In biomedicine, other small carbon molecules with unique physical and chemical characteristics, such as nanocarbons and nanodiamonds, are also developing and require more investigation.

## Virus-like particles: innovative nano-vehicles for theranostics in the future

Virus-like particles (VLPs) are multimeric nanostructures that self-assemble and range in size from 0.1 to 100 nm. They are produced when viral structural genes are expressed in heterologous systems (144). The VLPs are allowed to travel through the bloodstream, and their functional viral proteins are on the cell surface, which promote cell entrance and penetration (144). By targeting and infiltrating certain tumour cells through receptor-mediated endocytosis, VLP’s can encapsulate tiny molecules or drugs that may be used to treat cancer. Eventually, the encapsulated medicine will be released inside the cancer cell. The most amazing capacity is to avoid lysosomal degradation by escaping endosomes; this promotes medication availability and protects drugs in blood plasma (144).

The major drawback to using VLP as a drug delivery strategy is that, because of its viral proteinaceous particle and easy uptake by dendritic cells, it triggers an innate immune response (145). Nevertheless, after traditional chemotherapy failed, it provided a glimmer of hope for the treatment of TNBC (145). Moreover, improvements in medication absorption and biocompatibility may offset the aforementioned drawbacks. Human papillomavirus (HPV), bacteriophage, polyomavirus, Ebola, influenza, hepatitis E virus (HEV), and tobacco mosaic virus (TMV) are the sources of several VLPs (146). The bulk of VLPs exhibit natural tropism to sialic acids or heparin sulphates, which restricts their application as targeted nanocarriers. However, certain VLPs exhibit natural tropism to specific organs or tissues, such as HEV VLPs for the liver and hepatocytes (146).

The self-assembled Bacteriphage MS2 VLP, which is modified with SP94 peptide and encapsulated with doxorubicin, cisplatin, and 5-fluoro-uracil to selectively transport and kill human hepatocellular carcinoma (HCC) in the Hep3B cell line, is a classic example of VLPs as targeted therapeutic carriers (147). Dodecahedron, chemically coupled with the anticancer antibiotic Bleomycin (BLM) and produced from adenovirus (Ad3), or Db-BLM, causes ds-DNA breaks at lower concentrations, which kills transformed cells. Thus, the adaptability, cell-specific targeting, effective cell entry, absence of endosomal sequestering, multivalency, biocompatibility, massive encapsulation, and secure delivery mechanism of vector lambda proteins are responsible for their widespread use. Even with all of their benefits, VLPs as a medication delivery mechanism are still in their infancy and require validation using animal models (148).

## Conclusion and future prospect

Addressing the advantages of nanotechnology in TNBC treatment, researchers highlight the potential for targeted drug delivery systems that can enhance the efficacy of therapies while minimizing side effects. Adjuvant treatment, which includes hormone therapy (letrozole, tamoxifen) and chemotherapy (paclitaxel, eribulin), has a number of long-term adverse effects that lower the patient’s quality of life. Chemotherapy, which includes neoadjunant chemotherapy, anthracyclin, and taxane-based chemotherapy, is still the only choice for treating TNBC. A sophisticated, innovative, and successful therapy is required due to the 50% recurrence rate and 37% death rate, despite thorough and vigorous management.

The conjugation of multifunctional smart nanoparticles with therapeutic fluorophore allows them to penetrate various biological barriers, target, and penetrate cancer cells through a passive mechanism called the enhanced permeability and retention (EPR) effect. Ultimately, the controlled release of medication within the cancer cells is achieved. However, challenges such as the need for extensive safety evaluations and the complexity of manufacturing nanomaterials remain significant hurdles to overcome. There are a lot of limitations associated with conventional chemotherapeutic drugs, including non-specific targets, poor clinical results, chronic toxicity, poor solubility, low absorption, and potential drug resistance. Immunotherapy has produced new and useful treatment alternatives; however, using these immunostimulatory medications unmodified has led to drawbacks such as off-target effects and quick elimination. Immunotherapy based on nanoparticles has a lot of potential to overcome these challenges in order to increase the therapeutic effect and improve patient outcomes for TNBC patients.
